# A novel, robust, and portable platform for magnetoencephalography using optically-pumped magnetometers

**DOI:** 10.1162/imag_a_00283

**Published:** 2024-09-25

**Authors:** Holly Schofield, Ryan M. Hill, Odile Feys, Niall Holmes, James Osborne, Cody Doyle, David Bobela, Pierre Corvilain, Vincent Wens, Lukas Rier, Richard Bowtell, Maxime Ferez, Karen J. Mullinger, Sebastian Coleman, Natalie Rhodes, Molly Rea, Zoe Tanner, Elena Boto, Xavier de Tiège, Vishal Shah, Matthew J. Brookes

**Affiliations:** Sir Peter Mansfield Imaging Centre, School of Physics and Astronomy, University of Nottingham, University Park, Nottingham, United Kingdom; Cerca Magnetics Limited, Nottingham, United Kingdom; Université libre de Bruxelles, ULB Neuroscience Institute, Laboratoire de neuroanatomie et neuroimagerie translationelles, Brussels, Belgium; Department of Neurology, Hôpital Erasme, Hôpital Universitaire de Bruxelles, Université libre de Bruxelles, Brussels, Belgium; QuSpin Inc., Louisville, CO, United States; Department of Translational Neuroimaging, Hôpital Erasme, Hôpital Universitaire de Bruxelles, Université libre de Bruxelles, Brussels, Belgium; Centre for Human Brain Health, School of Psychology, University of Birmingham, Birmingham, United Kingdom

**Keywords:** optically-pumped magnetometer, OPM, magnetoencephalography, MEG, electrophysiology

## Abstract

Magnetoencephalography (MEG) measures brain function via assessment of magnetic fields generated by neural currents. Conventional MEG uses superconducting sensors, which place significant limitations on performance, practicality, and deployment; however, the field has been revolutionised in recent years by the introduction of optically-pumped magnetometers (OPMs). OPMs enable measurement of the MEG signal without cryogenics, and consequently the conception of “OPM-MEG” systems which ostensibly allow increased sensitivity and resolution, lifespan compliance, free subject movement, and lower cost. However, OPM-MEG is in its infancy with existing limitations on both sensor and system design. Here, we report a new OPM-MEG design with miniaturised and integrated electronic control, a high level of portability, and improved sensor dynamic range. We show that this system produces equivalent measures compared with an established OPM-MEG instrument; specifically, when measuring task-induced beta-band, gamma-band, and evoked neuro-electrical responses, source localisations from the two systems were comparable and temporal correlation of measured brain responses was >0.7 at the individual level and >0.9 for groups. Using an electromagnetic phantom, we demonstrate improved dynamic range by running the system in background fields up to 8 nT. We show that the system is effective in gathering data during free movement (including a sitting-to-standing paradigm) and that it is compatible with simultaneous electroencephalography (EEG). Finally, we demonstrate portability by moving the system between two laboratories. Overall, our new system is shown to be a significant step forward for OPM-MEG and offers an attractive platform for next generation functional medical imaging.

## 1 Introduction

Magnetoencephalography (MEG) measures the magnetic fields generated above the scalp by current flow through neuronal assemblies in the brain (Cohen, 1968). Mathematical modelling of these fields results in three dimensional images showing the spatial and temporal signatures of electrophysiological activity. MEG is a proven tool to investigate brain function, with applications in neuroscience and clinical practice (Baillet, 2017). In neuroscience, it can be used to measure evoked responses, neural oscillations, functional connectivity, and network dynamics—showing how the brain continually forms and dissolves networks in support of cognition. Clinically, MEG is used in epilepsy to localise the brain areas responsible for seizures as well as surrounding eloquent cortex (De Tiège et al., 2017). There are additional potential clinical applications ranging from the study of diseases that are common in childhood (e.g., measurement of the auditory evoked response latency in autism; Matsuzaki et al., 2019) to investigation of neurodegenerative conditions in older adults (e.g., cortical slowing in dementia; Gouw et al., 2021). MEG outperforms the clinical standard—electroencephalography (EEG)—in terms of both spatial precision (since magnetic fields are less distorted by the skull than the electric potentials measured by EEG) and sensitivity (since EEG is more effected by artefacts from non-neuronal sources—such as muscles) (Boto et al., 2019; Goldenholz et al., 2009). However, conventional MEG systems are based on cryogenically cooled (superconducting) sensors; this means systems have a high cost and are impractical for many applications, particularly compared with EEG. This has prevented widespread uptake of MEG systems.

In recent years, MEG has been revolutionised by the introduction of optically-pumped magnetometers (OPMs). (See Brookes et al., 2022; Schofield et al., 2023; Tierney et al., 2019 for reviews.) OPMs measure magnetic fields with a sensitivity similar to the sensors used for conventional MEG, but without the need for cryogenic cooling. They can also be microfabricated (Schwindt et al., 2007; Shah & Wakai, 2013; Shah et al., 2007, 2020) such that they are small and lightweight. This leads to multiple advantages. For example, sensors can be sited closer to the scalp surface (compared with cryogenic devices, as a thermally insulating gap is no longer required); this improves signal amplitude significantly (Boto et al., 2016, 2017; Iivanainen et al., 2017, 2019, 2020) and theoretical calculations suggest this can offer improved spatial resolution (higher than conventional MEG and EEG) (Nugent et al., 2022; Tierney et al., 2022; Wens, 2023). Arrays can be adapted to fit any head shape—from newborns to adults (Corvilain et al., 2024; Feys, Corvilain, Bertels, et al., 2023; Hill et al., 2019; Rier et al., 2024). Adaptability also means arrays can be designed to optimise sensitivity to specific effects (Hill et al., 2024) or brain areas (Lin et al., 2019; Tierney, Levy, et al., 2021). As the sensors move with the head, participants can move during recordings (assuming background fields are well controlled) (Holmes et al., 2018, 2019, 2023; Rea et al., 2021). This enables the recording of data during novel tasks (Boto et al., 2018; Rea et al., 2022) or even epileptic seizures (Feys, Corvilain, Bertels, et al., 2023; Hillebrand et al., 2023). The adaptability to different head size/shape coupled with motion robustness (Feys & De Tiège, 2024) means that, like EEG, OPM-MEG systems are wearable. However, unlike EEG, sensors do not need to make electrical contact with the head, making OPM-MEG more practical than EEG in terms of both patient friendliness and set-up time. Finally, even at this early stage of development, OPM-based systems are becoming cheaper to buy and run than conventional MEG devices. These significant advantages could—in theory—lead to OPM-MEG becoming the method of choice for electrophysiological measurement, potentially replacing EEG as a clinical tool for some applications.

Despite its promise, OPM-MEG remains in its infancy. The optimum system design has not yet been settled and the OPMs themselves remain limited in performance with higher noise compared with cryogenic sensors, lower bandwidth (though it is adequate for most MEG signals of interest), and much smaller dynamic range. To date, most published OPM-MEG studies have used systems composed of multiple independent sensors which are joined together and synchronised to form an array. Such systems work (e.g., Boto et al., 2018; Corvilain et al., 2024; Feys et al., 2022; Hill et al., 2020; Iivanainen et al., 2019) but their electronic architecture is complex and can be prone to failure. In addition, whilst the OPM-MEG helmet is lightweight, electronics racks are large, cumbersome, and must be kept outside the magnetically shielded room to limit magnetic interference. As a result, long cables must pass through waveguides in the MSR to the electronics. Such cabling can be prone to interference. Moreover, when systems have large sensor counts, cabling becomes cumbersome with large number of wires trailing from the subject. While this is fine for static systems, if the aim is to allow the subject to move freely (even to walk around a room; Holmes et al., 2023), having heavy cabling draped around a participant is impractical—particularly for patients.

At a technical level, OPMs already have high sensitivity; even though noise levels are higher than conventional sensors (Boto et al., 2022), proximity to the scalp enables improved signal strength (Hill et al., 2024). However, perhaps their biggest limitation is dynamic range. This is because the sensor output is only a linear function of magnetic field within a very narrow range of field—approximately -1.5 nT to +1.5 nT for rubidium OPMs. Such a narrow range is problematic; whilst the MEG signal itself is small relative to this window, even in magnetic shields environmental field fluctuations can be much larger. The problem is even more complicated if the head is allowed to move, since even in the absence of time-varying environmental fields, movement in a static field can take sensors outside their dynamic range. This problem is notionally solved by operating sensors in “closed-loop” mode, where sensors use negative feedback such that any changes in field at the sensor are compensated by on-board sensor electromagnetic coils. However, closed-loop operation is complicated since fields oriented in all three directions relative to the sensor affect the linearity of the response (Schofield et al., 2023; Tierney et al., 2019), meaning closed-loop operation is required on three axes.

These limitations mean that extant OPM-MEG systems are not yet the “final product” and there remains significant scope for development. Here, we demonstrate a new platform with a miniaturised electronic control system that solves many of the practical limitations associated with the current generation of instrumentation. In what follows, we report a study demonstrating the equivalence of our new system to established OPM-MEG hardware. We use an electromagnetic phantom (a device that makes “brain-like” magnetic fields) to confirm that closed loop operates as intended. We exploit closed-loop measure brain activity as participants move freely—including a sitting-to-standing task. We exploit the miniaturised nature of the electronics by transporting our system between two laboratories and finally we pair our new system with EEG, demonstrating that we can acquire simultaneous OPM-MEG and EEG data.

## 2 Materials and Methods

### 2.1 OPM-MEG system comparison

#### 2.1.1 Systems and data acquisition

We initially aimed to compare two OPM-MEG systems. Both comprised 64 triaxial OPM sensors (QuSpin Inc., Colorado, USA) each capable of measuring magnetic field in 3 orthogonal orientations, enabling data collections across 192 independent channels. The sensor design is well documented (Boto et al., 2022; Shah et al., 2020) and will not be repeated in detail here. Briefly, each sensor head is a self-contained unit incorporating an ^87^Rb vapour cell, a laser for optical pumping, on-board electromagnetic coils for field control within the cell, and two photodiodes for signal readout. A beam splitter splits the laser output and associated optics projects two orthogonal beams through the cell, to enable triaxial field measurement. The median noise floor of the sensors was expected to be ~15 fT/sqrt(Hz) in the 3–100 Hz range. This is higher than the noise floor of typical single or dual axis OPMs due to the requirement to split the laser beam for triaxial measurement (Boto et al., 2022).

**Fig. 1.**
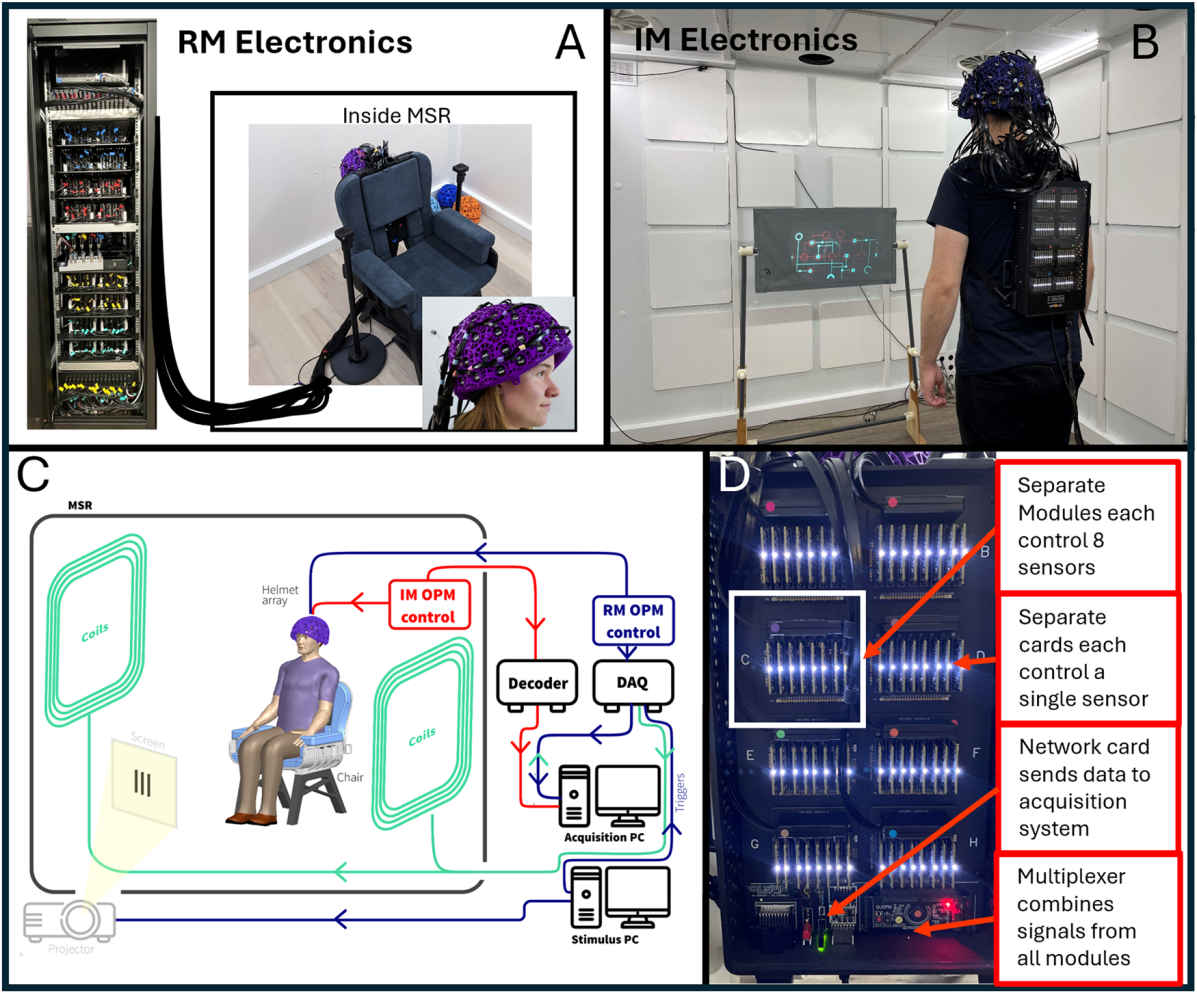
OPM-MEG systems: (A) Rack-mounted (RM) OPM-MEG system; sensor heads controlled via an electronics rack outside the MSR. (B) Integrated miniaturised (IM) OPM-MEG system; all control and acquisition electronics contained on within a backpack worn by the subject. (C) System schematic—valid for both systems with the major difference being the OPM electronics: Red pathways show the IM system, blue the RM system. (D) Photograph of the electronics for the integrated miniatured system.

Sensors from both systems were mounted in identical 3D-printed helmets (Cerca Magnetics Limited, Nottingham, UK), ensuring that the array geometry was the same for all measurements (see Fig. 1A—inset photo). The arrays were housed in a magnetically shielded room (MSR) comprising four mu-metal layers and one copper layer to attenuate DC/low-frequency and high-frequency magnetic interference fields, respectively (Magnetic Shields Limited, Kent, UK). The MSR walls were equipped with degaussing coils to reduce remnant magnetisation prior to a scan. The MSR was also equipped with a matrix coil (Holmes et al., 2023) and a fingerprint coil (Holmes et al., 2019)—both capable of active field control (Cerca Magnetics Limited, Nottingham, UK). A single “acquisition” computer was used for OPM-MEG control and data acquisition; the experimental paradigms (along with associated temporal markers (“triggers”) delineating the time at which stimuli were presented to the subject) were controlled by a second “stimulus” computer. Visual stimuli were presented via projection through a waveguide onto a back projection screen positioned ~100 cm in front of the subject. We used an Optoma HD39 Darbee projector with a refresh rate of 120 Hz. Schematics of both systems are shown in Figure 1C.

The two OPM-MEG systems differed in terms of their electronics control.

- **Established “Rack-mounted” (RM) System:** In the case of our established system (Fig. 1A), each sensor head was connected by a lightweight (2.2 gm^-1^) ribbon cable, 90 cm in length, to a backpack. The backpack housed 64 junction boxes in which the ribbon cables were connected to more robust cables (40 gm^-1^; 550 cm in length). Of these larger cables, 64 pass from the backpack, through waveguides in the MSR, and connect to an electronics control rack positioned outside the MSR. Each sensor head is controlled independently by an electronics unit which provides all control signals (including temperature modulation (via proportional-integrative-derivative control)) for the vapour cell and laser as well as on-board sensor coil control (including the modulation signals required for directional sensitivity (Cohen-Tannoudji et al., 1970)). The output of each sensor electronics unit includes three analogue signals which represent the three orthogonal fields measured by the sensor. These 192 separate signals are fed into a single data acquisition system (cDAQ 9178, National Instruments), where they are digitised synchronously and sent to the acquisition computer. The DAQ also has 16 additional channels for digital and/or analogue triggers as well as peripheral analogue signals (here we only needed 6 triggers for the paradigm). The independently controlled sensors are synchronised via a shared 923 Hz sinusoidal reference signal for demodulating the sensor lock-in amplifier to generate the output, and an externally generated 307 kHz sinusoid heater signal fed into each unit. The rack is powered by two 300 W power supplies. The rack is 2.02 x 0.6 x 0.6 m^3^ and weighs approximately 240 kg; the total weight of cabling between the backpack and the electronics rack is 14 kg.
- **“Integrated Miniaturised” (IM) system:** Here, all control electronics are housed in a backpack which is worn by the subject (Fig. 1B) (*Neuro-1-electronics*, QuSpin, Colorado, USA—https://quspin.com/neuro-1-an-integrated-sensor-system-for-opm-meg/). Sensor heads were connected to the backpack by a ribbon cable: 2.2 gm^-1^ and 90 cm in length. Sensors are grouped into modules, with a single module controlling up to eight sensors; each sensor is controlled by a single electronics card which—like the electronics units in the RM system—provides control signals for all on-board sensor components. Unlike the RM system, this device is capable of three-axis closed-loop operation in which the magnetic field is measured at each OPM along all three orthogonal axes, and the electronics effects a negative feedback loop, whereby currents are applied to the on-board sensor coils to maintain zero field at the vapour cell. This linearises the OPM response to external fields and (theoretically) extends the dynamic range to ±8 nT. The bandwidth of the OPMs is 0–135 Hz for both open- and closed-loop operation. Each electronics card contains its own DAQ and the outputs from all modules are digital. Those outputs are sent to a multiplexer and then to a network card. The backpack is connected via ethernet to a decoder positioned outside the MSR (DAQ—sbRIO9637, National Instruments). This integrates the MEG signals with triggers sent from the acquisition PC, samples all signals synchronously, and passes them to the acquisition PC via an ethernet connection. (Note: the system contains three BNC inputs and a parallel port for digital triggers and eight analogue-to-digital converters for peripheral signals, though again only six channels were required for the experiments presented here.) The only other connection to the backpack is a power cable. The backpack is 0.36 x 0.2 x 0.06 m^3^ and weighs approximately 1.8 kg. The total weight of cables between the backpack and the decoder is 1.3 kg.

Data were recorded from our RM system at 1200 Hz, and from our IM system at 375 Hz (this was the same for all experiments, except the “Sitting-to-standing task” which was sampled at 1500 Hz). The difference in sampling frequency was to reduce the size of the data files for most of the IM data recordings, as these were recorded on a laptop for added portability. For all recordings, participants were seated comfortably on a patient support located in the centre of the MSR. Prior to the recording, the inner walls of the MSR were degaussed, and the fingerprint coils energised using pre-determined currents. This ensured that the coils generated magnetic fields in the region surrounding the subject’s head that were equal and opposite to the fields typically observed in the MSR (these had been determined based on field measurements made across multiple previous experimental sessions (Rhodes et al., 2023)). The field surrounding the participant’s head is typically reduced from ~3 nT to ~0.7 nT using this method (Rea et al., 2022; Rhodes et al., 2023). Participants were free to move throughout the recording. For our IM system, data for this experiment were recorded in *open-loop* mode.

We used optical scanning to determine how the helmet was positioned on the subject’s head. Immediately following MEG data acquisition, a 3D digitisation of the participant’s head (with the helmet in place) was acquired using a 3D structured light scan (Einscan H, SHINING 3D, Hangzhou, China). The 3D surface of the subject’s face was extracted from this scan and matched to the equivalent surface taken from a T1-weighted anatomical magnetic resonance image (MRI). This enabled a co-registration of the helmet to brain anatomy (Hill et al., 2020; Zetter et al., 2019). Following this, detail of the precise locations and orientations of the sensors within the helmet (generated by the 3D printing of the helmet itself) was added to give a complete description of sensor locations/orientations relative to anatomy.

#### 2.1.2 Experimental paradigm

Our first aim was to demonstrate that our new IM system had performance similar to the established RM system (Rea et al., 2022; Rhodes et al., 2023; Rier et al., 2023, 2024). To this end, we scanned the same individuals multiple times using both systems (see Fig. 1A, B) and compared results. Two healthy participants (both male, ages 28 and 43 years) took part in the study, which was approved by the University of Nottingham Faculty of Health Sciences (UoNFHS) Research Ethics Committee (approval number H16122016). Each participant was scanned six times in each system over a period of 3 days (the order of scanning was counterbalanced). The scanning session was repeated at the same time each day for each participant (one participant session in the morning and one in the afternoon). The experiment consisted of a visuo-motor paradigm (Hill et al., 2022), containing three trial types:

1) **Circles trials:** A visual stimulus (a central, inwardly moving, maximum-contrast circular grating) was presented. The grating subtended an angle of 6° and was displayed for a duration of 1 s. This was followed by a (jittered) baseline period lasting 1.25 ± 0.2 s where a central cross was displayed. There were 60 circles trials per experiment. This stimulus is known to induce gamma oscillations in visual cortex (e.g., Hill et al., 2022; Hoogenboom et al., 2006; Iivanainen et al., 2020).
2) **Faces trials:** Visual stimulation was a photograph of a face, displayed on screen for a duration of 0.5 s, followed by a jittered rest period of duration 1.25 ± 0.2 s (during which a fixation cross was shown). A total of 120 faces trials were used. This task generates evoked responses in primary visual and fusiform areas (e.g., Bentin et al., 1996; Halgren et al., 2000; Hill et al., 2022; Taylor et al., 2001).
3) **Catch trials:** Here, a cartoon character was displayed for 0.8 s. A total of 25 catch trials were presented, and upon presentation, the subjects were asked to press a button with the index finger of their right hand. Such movements elicit robust modulation of beta oscillations in primary sensorimotor cortices (Pfurtscheller & Lopes da Silva, 1999).

The trial types were pseudo-randomised across the experiment, and the total experimental duration was 396 s. Prior to all sessions, empty room data were also collected using both systems.

#### 2.1.3 Data analysis

Channel data were initially inspected by computing spectral densities (estimated by dividing the recording into segments and then averaging adjusted periodograms of these segments). Any channels for which the mean noise in the 60–80 Hz band was >30 fT/rt(Hz) or <7 fT/rt(Hz) were automatically removed. A trial-by-trial analysis was also carried out and bad trials were defined as those with variance greater than 3 standard deviations from the mean trial variance, and automatically removed. Data were also inspected visually, and any obvious noisy channels or trials were removed. Notch filters at the powerline frequency (50 Hz) and two of its harmonics, as well as a 1–150 Hz 4th order Butterworth band pass filter were applied. Finally, homogeneous field correction (HFC) was applied to reduce interference caused by distant sources (Tierney, Alexander, et al., 2021). We used a beamformer spatial filter (Robinson & Vrba, 1999) to construct either pseudo-T- or pseudo-Z-statistical images showing the spatial signature of task-induced change in source power or amplitude. We also used a beamformer to construct timecourses of electrophysiological activity at locations of interest (derived from locations informed by the statistical images—termed a “virtual electrode”). In all cases, the forward model was based on a single shell volume conductor model (Nolte, 2003). Processing was different for the three trial types:

- **For circles trials**, data were segmented to 0 to 2 s windows (relative to the onset of the circle) and filtered to the 52 Hz to 65 Hz frequency band (as the circular grating is known to elicit a narrow-band response (Hoogenboom et al., 2006; Iivanainen et al., 2020)). A covariance matrix and beamformer weights were constructed using filtered data from all circles trials; covariance matrices were regularised using a regularisation parameter equal to 1% of the maximum eigenvalue of the unregularised matrix (this is the case for all beamformer images derived in this paper). To make the pseudo-T-statistical image, we contrasted power in the 0 to 0.6 s (active) window to power in the 1.1 to 1.7 s (control) window, deriving a pseudo-T-statistic for voxels on a regular 4-mm grid covering the brain. To generate a virtual electrode, beamformed data local to the peak in the pseudo-T-statistical image were Hilbert transformed and the analytic signal was derived. The absolute value of this signal was then used to give the envelope of oscillatory amplitude (*Hilbert envelope*) in the gamma band, which was trial averaged.
- **For faces trials**, data were segmented to 0 to 1.5 s windows (relative to the presentation of a face) and filtered to the 2 Hz to 40 Hz band. Covariance and weights were constructed using data from all faces trials. To compute the evoked response, we first used a beamformer to reconstruct a virtual electrode at an anatomically defined point in the fusiform cortex (selected according to the automated anatomical labelling atlas (Gong et al., 2009; Hillebrand et al., 2016; Tzourio-Mazoyer et al., 2002)). Beamformed timecourses were averaged across trials, giving the evoked response. For the peak in the evoked response (at ~170 ms), we generated a pseudo-Z-statistical image (which contrasts beamformer projected source power (at a single point in time) to the estimated noise power (Van Veen et al., 1997)). This allowed us to assess spatial signature of the evoked response, for each experimental run.
- **For catch trials**, data were segmented to -0.3 to 1.7 s windows (relative to the button press) and filtered to the beta (13 Hz to 30 Hz) band. Covariance and weights were constructed using data from all catch trials and the pseudo-T-statistical image contrasted power in the -0.3 to 0.3 s window to power in the 0.8 to 1.4 s window. A virtual electrode was generated for a location derived from the pseudo-T-statistical image, using the Hilbert envelope to show the time evolution beta band oscillatory amplitude.

In all cases, data from both systems and all experiments were processed in the same way and results compared. Note also that we used a 4th order Butterworth filter for all frequency filtering.

### 2.2 Closed-loop operation

Our second aim was to demonstrate that the closed-loop enabled operation of our IM system in the presence of large background magnetic fields. For this we used a two-step approach, first employing a phantom to make known magnetic fields, and then moving to a naturalistic experiment in a human participant.

#### 2.2.1 Phantom study

We used a dry-type current dipole phantom (Holmes et al., 2023; Oyama et al., 2015; Rier et al., 2023) to generate magnetic fields mimicking brain activity (henceforth called the *phantom field*). The phantom comprised a triangular electromagnetic coil (isosceles, 5-mm base and 45-mm height; made from a single turn of 0.56-mm diameter enamelled copper wire. The ends of the wire were twisted to avoid any stray magnetic fields.) The phantom was enclosed in a Perspex cylinder and glued to an empty OPM sensor casing allowing it to be fitted inside the OPM helmet with a known position and orientation relative to the sensor array. We performed two experiments.

- ***Response linearity in zero background field**:* We first tested whether closed-loop operation had any effect on measurements being made in “zero” background field (practically this means a background field at each OPM of <700 pT, well within normal (open loop) operational range). A sinusoidal current of frequency 17 Hz was applied to the phantom for 2 s followed by 1 s at zero current, to mimic experimental trials. The amplitude of the current waveform was varied between 9 values [0.01, 0.02, 0.05, 0.08, 0.1, 0.2, 0.5, 0.8, 1] mA, corresponding to an expected magnetic field between 20 and 200 pT at the OPM with the largest response. This process was repeated eight times, and a trigger signal was used to mark periods in the data when the phantom was active. The experiment took ~7 min and was repeated twice, once with sensors in open-loop mode, and a second time with sensors in closed-loop mode. To check linearity, we took the sensor outputs (for all channels) for both datasets, segmented data into 2-s periods where the phantom was active using the trigger signal, and applied a Fourier transform to compute a magnitude spectrum. We extracted the magnitude of the 17-Hz phantom signal for each channel, for all dipole amplitudes and trials (i.e., 64 measurements per channel per condition), and plotted all the open-loop sensor amplitudes against the same measures made using the closed loop. We expected that in this (zero-background-field) case, the values would show a linear relationship, meaning that closed-loop operation is not affecting the measurement.
- ***Sensor response in non-zero background fields**:* In the absence of closed-loop sensor operation, we would expect the presence of a large background field to affect the OPM response according to the solution to the Bloch equations which govern the polarisation of the rubidium gas (see Cohen-Tannoudji et al., 1970 for a complete description)—in general, as background field (in any orientation relative to the sensors) is increased, one would expect the individual OPM response to the phantom field to decrease in amplitude. To investigate this, we used the matrix coil (Holmes et al., 2023), embedded in the walls of the MSR, to generate a controlled *background field*; this was uniform across the volume occupied by the OPM helmet and oriented vertically. We also generated a phantom field using the same current waveform as before, but at a single amplitude—1 mA. We applied the waveform five times in zero applied background field, then stepped the background field up from 0 nT to 8 nT in 81 steps of 0.1 nT, repeating the measurement of phantom field waveforms after each step up in background field. The whole experiment took around 20 min and was repeated twice with sensors in open loop and closed loop. We segmented the data and computed the magnitude spectrum as before, again plotting corresponding open-loop versus closed-loop measurements of the phantom field, for all trials and all values of background field (405 measurements per channel per condition). Here we expected that closed-loop operation would result in faithful reconstruction of the expected field, whereas with open-loop operation, the measured field would diminish with increasing background field, and, unlike the previous experiment, there would be no linear relationship between the two operational modes.

#### 2.2.2 Sitting-to-standing task

As a further demonstration of closed-loop operation, we recorded data during a naturalistic task. A single participant (male, aged 28 years) took part in the study which was again approved by the UoNFHS Research Ethics Committee (H16122016). In our “sitting-to-standing” task, trials lasted 8 s and were cued by two alternating auditory stimuli (either a 1400-Hz tone or a 1000-Hz tone, both lasting 1 s). On hearing the 1400-Hz cue, the participant moved from sitting to standing; on the 1000-Hz cue, the participant went from standing to sitting. Whilst moving, they performed abductions of their right index finger. As the head moves, it will experience a changing magnetic field; we purposely did not degauss the MSR or engage active field compensation prior to the measurement, to maximise the field change that would be experienced by the sensors. (We expected a field change of order 2–3 nT—sufficient to take OPMs operating in open loop outside their dynamic range.) With closed-loop operation, we hypothesised that the beta band modulation induced by the movements would be successfully recorded, despite the large shift in background field.

Data were processed using a pipeline similar to that described above: Following bad channel/trial rejection, data were segmented to 0 to 8 s windows (relative to the auditory cues) and filtered to the 13 Hz to 30 Hz band. Covariance and beamformer weights were constructed using data from all trials and a pseudo-T-statistical image contrasted oscillatory power in the 2 to 3 s window to that in the 6.5 to 7.5 s window. To examine signal dynamics, we constructed a time frequency spectrogram (TFS). The pre-processed data were frequency filtered into the 1–150 Hz band and data covariance and beamformer weights estimated. A virtual electrode was generated for a location derived from the peak in the pseudo-T-statistical image. The resulting (broadband) beamformed data were filtered into a set of overlapping frequency bands (4th order Butterworth filter), and the Hilbert envelope was computed for each band; this was averaged across trials and concatenated in frequency. Baseline correction was applied by subtracting the time average of oscillatory amplitude, for all bands, in the control window. The spectrum was normalised by oscillatory power in the control window. The result was a TFS showing relative change in oscillatory amplitude, for all frequencies, over time.

### 2.3 Demonstrating portability

One advantage of our IM system is that the small and lightweight electronics makes it transportable. Our third aim was to provide a proof-of-principle of this portability by moving the system between two laboratories and recording data in a group of healthy volunteers. The two sites differed as follows:

- **Site 1: University of Nottingham, Nottingham, UK:** The system was housed in an MSR (Magnetic Shields Limited) with internal dimensions 3 x 3 x 2.4 m^3^, and walls comprising four layers of mu-metal and a single copper layer. The room was equipped with degaussing, and both matrix and fingerprint coils for active field control. All experiments were approved by the UoNFHS ethics committee (H16122016).
- **Site 2: Hôpital Erasme, Bruxelles, Belgium:** The system was housed in a compact MSR (Magnetic Shields Limited) with internal dimensions 1.3 x 1.3 x 2.4 m^3^, and walls comprising two layers of mu-metal and a single layer of copper. The room is equipped with degaussing coils and a “window coil” for active field control (Holmes et al., 2022). Comité d’Ethique hospitalo-facultaire Erasme approved this study (approval number P2019/426/B406201941248) and participants gave written informed consent.

The same IM system (in closed-loop mode) was used in both laboratories, and was transported between sites in two suitcases, via rail. Five individuals took part in the experiment at each site (two females and three males aged 25–33 years at site 1 and one female and four males aged 27–47 years at site 2).

The experimental paradigm was a motor task; a single trial comprised 3 s of a right index finger abduction, followed by 3 s rest. A total of 100 trials were collected over 2 runs of 50 trials. All data were collected at a sampling rate of 375 Hz. Data pre-processing was as described above, except a template brain warping method (Rier et al., 2024) was used to create a “pseudo-MRI” for each subject as opposed to an anatomical MRI. Pseudo-T-statistical beamformer images were derived for the beta band (13 Hz to 30 Hz), by contrasting active (0.5 s to 1.5 s) and rest (3.5 s to 4.5 s) windows. TFSs were derived from locations of interest derived from peaks in the images using the method described above.

### 2.4 Concurrent OPM-MEG EEG

EEG remains the most common clinical metric of brain function, and has proven utility in disorders including epilepsy, dementia, head injury, sleep disorders, and encephalitis (Ding & Yuan, 2013). Previous work suggests that OPM-MEG can outperform EEG in terms of sensitivity, spatial resolution, and practicality (Boto et al., 2019). However, in practice it would be advantageous to acquire data from both modalities simultaneously; this would not only enable integration of signals (which has advantages (Aydin et al., 2015; Feys, Wens, et al., 2023; Yoshinaga et al., 2002)), but also allow clinicians to benefit from the improved performance of OPM-MEG whilst still having access to EEG data (Feys, Wens, et al., 2023). The use of simultaneous scalp EEG during MEG recordings in epileptic patients is mandatory according to international clinical practice guidelines (Bagić et al., 2011, 2023; Feys, Wens, et al., 2023). It allows for a better classification of physiological versus pathological brain activities (Rampp et al., 2020). It also provides a truly complementary measure to MEG, such that neural sources that are “silent” in one modality can be detected in the other modality (Mosher & Funke, 2020). The simultaneous collection of OPM-MEG and EEG data has been demonstrated previously (Boto et al., 2019; Feys, Corvilain, Van Hecke, et al., 2023)—here our aim was simply to show that it was also possible using our IM system.

To this end, we employed a 64-channel MEG-compatible EEG system (Brain Products GmbH, Munich, Germany) comprising an EEG cap (with passive, MEG compatible, AgCl electrodes), signal amplifiers, a power pack, and a data acquisition laptop. During data acquisition, the ground electrode was AFz and the reference electrode was FCz. In total, 63 channels were attached to the scalp and 1 to the subject’s back to measure the electrocardiogram (ECG). Conductive gel (Abralyte 2000) was used to connect electrodes to the head with all impedances kept below 10 kΩ. Our 192-channel OPM-MEG helmet was placed over the top of the EEG cap and was otherwise operated (in closed-loop mode) as described above. Triggers from the stimulus PC were split so that simultaneous markers appeared in the OPM-MEG and EEG data, allowing data alignment. Five subjects took part in the experiments (one female and four males, age 27–33 years) which were approved by the UoNFHS Research Ethics Committee (H16122016). A single recording comprised 60 circles trials and 80 faces trials—though here we only analysed the circles trials, in which participants were asked to complete a finger abduction with their right finger whilst a circle was on the screen. The experiment was carried out twice per subject, once when the participant was asked to remain still and once when they were asked to make natural head movements (head movements were tracked via infrared markers placed on the helmet, tracked via OptiTrack (Natural Point Inc.) motion tracking cameras.

Two 3D digitisations using structured light (Einscan H, SHINING 3D, Hangzhou, China) were taken: one of the participant wearing the OPM helmet (so that MEG co-registration could be carried out, as previously described) and one of the EEG cap. Electrode positions were found from the 3D scan of the EEG cap. These were then matched to a layout of the sensors in the cap by first manually point-matching specific electrodes, and then using an iterative closest point (ICP) algorithm. The electrode array was then co-registered to the MRI by matching fiducial points on the digitisation and MRI.

OPM-MEG processing was as described above. EEG data preprocessing comprised removal of bad channels and trials. For the removal of bad EEG channels and trials, raw timecourse data were evaluated and any channels/trials that were (visually) noisier than average were rejected. Following this, data from remaining circles trials were segmented, concatenated, and filtered (independently) to both the beta (13–30 Hz) and gamma (30–80 Hz) bands. We constructed an EEG forward model using a three-shell boundary element model implemented in Fieldtrip (Oostenveld et al., 2011). We then processed the data from both EEG and MEG using the same beamformer approach as was used in OPM-MEG (forward model and beamformer code available from https://github.com/SCColeman/EEG_beamformer). Covariance matrices were generated using band limited data. Pseudo-T-statistical images were derived on a 4-mm grid spanning the whole brain volume. For visual gamma effects, we contrasted the 0.2 s to 0.8 s active window with the 1.2 s to 1.8 s control window; to look for sensorimotor beta modulation, we contrasted a 0.5 s to 1.0 s active window with a 1.5 s to 2.0 s control window. For the beta modulation, we derived TFSs from the peak location found independently using both EEG and OPM-MEG data. For the visual gamma effects in both the EEG and OPM-MEG, TFSs were derived from an anatomically defined location in the visual cortex (selected by taking the centre of mass of the calcarine region according to the automated anatomical labelling atlas (Gong et al., 2009; Hillebrand et al., 2016; Tzourio-Mazoyer et al., 2002)). This was due to inconsistency in the localisation of the gamma response in EEG. This process was repeated in OPM-MEG for consistency.

## 3 Results

### 3.1 Notes on system operation

Our IM system had a representative median (across sensors) noise floor of 13.8 fT/sqrt(Hz) (in the 3–100 Hz band); the 5th and 95th percentile measures were 10.4 fT/sqrt(Hz) and 19.3 fT/sqrt(Hz), respectively. This was compared with a median of 11.8 fT/sqrt(Hz) for our RM system, with the 5th and 95th percentile measures 8.8 fT/sqrt(Hz) and 16.6 fT/sqrt(Hz). (Noise spectra from both systems, with and without HFC and (for the IM system) in open and closed loops, are shown in Supplementary Fig. S1.) We acquired data in 42 experiments using our IM system (2 subjects x 6 comparison scans; 4 phantom experiments; 1 sitting-to-standing task; 2 sites x 5 subjects x 2 runs cross-site study, and 5 OPM-MEG/EEG experiments). Across all 42 experiments, we lost on average 3 ± 5 channels (mean ± standard deviation). These lost channels were typically due to an OPM sensor head becoming detached from the ribbon cable. The system typically took ~60 s to start the 64 sensors (this procedure includes heating all sensors, locking laser frequencies (and temperatures), and optimising control parameters). Zeroing the magnetic field (using on-board sensor coils) and calibrating each sensor took a further ~60 s. The total system set-up time (including degaussing and field nulling) was approximately 3 min. This was a set-up time similar to the RM system.

### 3.2 OPM-MEG system comparison

**Fig. 2.**
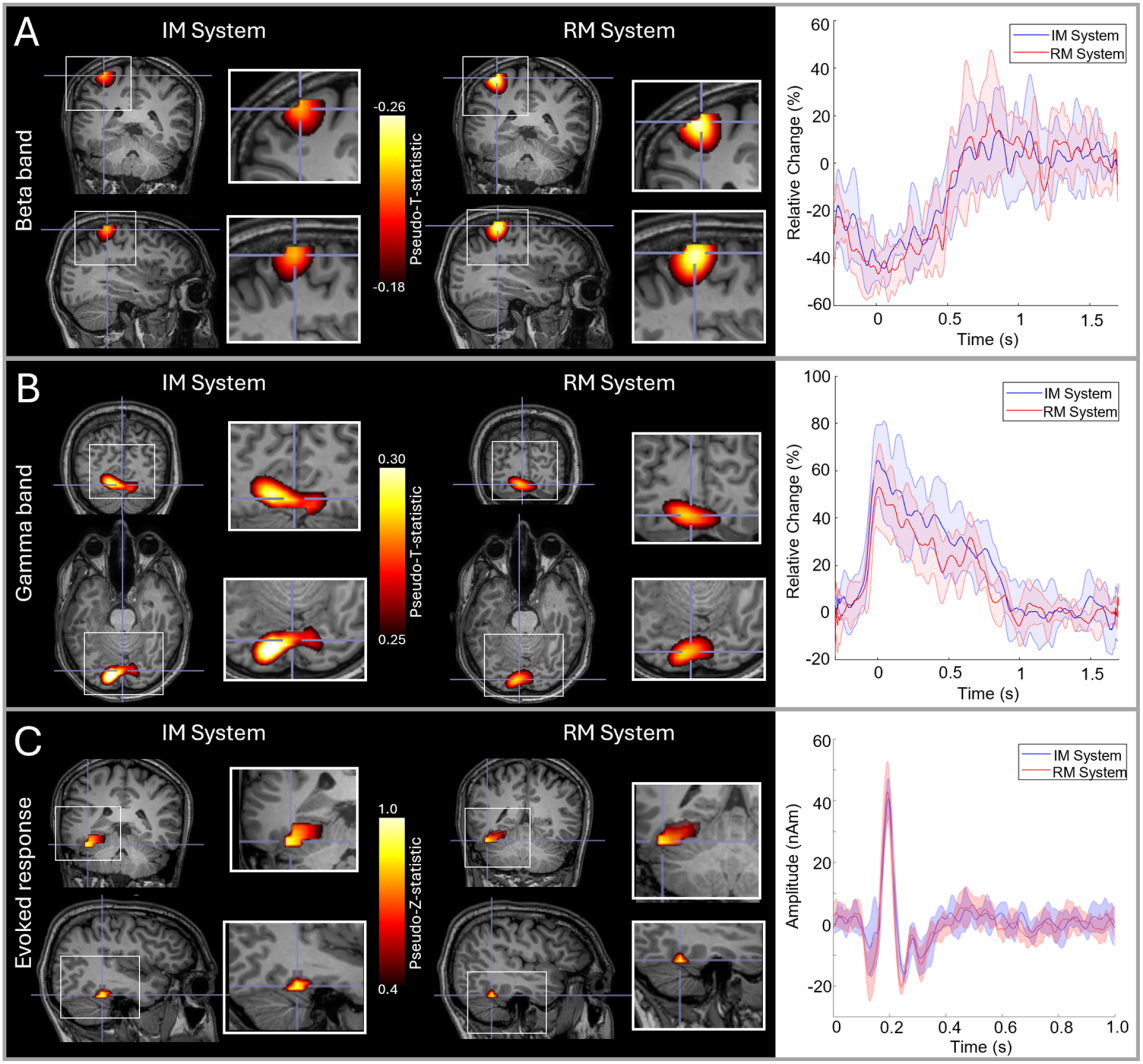
RM and IM system comparison: (A) Beta band responses to a finger movement; in the images on the left the overlay shows the location of maximum beta modulation and the timecourses on the right show the time evolution of beta band amplitude. (B) Gamma responses to visual stimulation; images show the locations of gamma modulation and timecourses show evolution of gamma band amplitude. (C) Evoked responses to face presentation; images show the location of the highest evoked power and timecourses show trial-averaged evoked responses. In all three cases, data are averaged across six runs; images from both systems are shown, and in the timecourse plots, red represents the RM system, blue the IM system, and the shaded areas represent standard deviation over runs. Data are shown in a single subject (S2). Data from the second subject are shown in Supplementary Figure S2.

Figure 2 shows the results of the comparison between our RM and IM systems. Results for a single subject are shown (averaged across all six runs); an equivalent figure for the second subject is provided in Supplementary Figure S2. Panel A shows beta modulation during the button press. In both systems, the largest beta modulation was localised to the left primary sensorimotor cortices (due to movement of the right index finger) and the timecourse shows a clear movement-induced reduction in beta amplitude, as expected. Figure 2B shows gamma modulation during presentation of the circles stimuli. Here, the largest stimulus-induced increases were in primary visual areas and the expected increase in gamma amplitude during stimulus presentation is observed. Figure 2C shows evoked responses to face presentation. The images show the spatial signature of the evoked response, at a latency of ~170 ms, which was predominantly in the fusiform area.

To quantify the spatial agreement between runs, we measured the Euclidean distance between the voxels showing the largest task-induced signal modulation. This was done for all three responses (beta, gamma, and evoked) and in three ways:

1) **Individual runs—between system:** In a single participant, for each measurement (beta, gamma, and evoked response), we had six pseudo-T/Z-statistical images for each system. This gives 36 possible comparisons between systems (run 1–to run 1; 1–2; 2–2, etc.). For each, we found the Euclidean distance between peak voxels. We then found the mean and standard deviation of these values.
2) **Individual runs within system:** From the 6 runs in a single system, there are 15 possible comparisons (i.e., runs 1-to-2, 1-to-3, 2-to-3, etc.). For each within-system comparison, we again measured Euclidean distance between the peak voxels, computing the mean and standard deviation.
3) **Averaged runs:** We measured the Euclidean distance between the peaks in the pseudo-T/Z-statistical images averaged across runs in the same system.

The results of these three analyses, which were computed for each subject, are shown in Table 1. The spatial discrepancies between runs tended to be larger for the gamma and evoked experiments than for the beta experiments (see discussion). However, in all cases, the within-system comparison is not notably different to the between-system comparison, suggesting that there is no major difference in spatial localisation between systems.

**Table 1.** Spatial robustness.

Distance	Individual runs (mm)						Averages (mm)	
	RM to IM		Within RM		Within IM		RM to IM	
Response	S1	S2	S1	S2	S1	S2	S1	S2
Beta	11 ± 8	8 ± 4	13 ± 9	9 ± 3	8 ± 4	9 ± 5	7	2
Gamma	34 ± 14	17 ± 8	31 ± 23	20 ± 8	30 ± 20	17 ± 8	14	3
Evoked	20 ± 13	22 ± 15	17 ± 11	16 ± 11	23 ± 12	30 ± 17	11	7

All values represent Euclidean distances in millimetres between peaks in pseudo-T-statistical images. Comparisons are made between systems for individual runs, within systems for individual runs, and between systems for the averages across runs, for two subjects (S1 and S2).

To quantify the temporal agreement between runs, we used Pearson correlation between the reconstructed timecourses of either induced (beta/gamma) or evoked activity. Again, we used three measures:

1) **Individual runs—between system:** We computed all 36 possible values of correlation between all pairs of experiments in the RM and IM system.
2) **Individual runs within system:** We calculated 15 correlation coefficients representing the similarity of experimental timecourses within each system (i.e., 30 measures in total).
3) **Averaged runs:** We calculated the correlation between timecourses averaged across runs.

As before, these calculations were carried out for each subject and paradigm separately. Results are shown in Table 2. First, note that all values of correlation are high (on average 0.77 for individual runs and 0.91 for averaged runs), indicating that both systems are reliable. Again, we found little difference between the within-system correlations (mean = 0.76) and between-system correlations (mean = 0.77).

**Table 2.** Temporal robustness for subject 1 (S1) and subject 2 (S2).

Correlation	Individual runs						Averages	
	RM to IM		Within RM		Within IM		RM to IM	
Response	S1	S2	S1	S2	S1	S2	S1	S2
Beta	0.81 ± 0.05	0.75 ± 0.06	0.81 ± 0.03	0.78 ± 0.05	0.80 ± 0.06	0.72 ± 0.07	0.96	0.96
Gamma	0.67 ± 0.08	0.81 ± 0.07	0.67 ± 0.08	0.79 ± 0.05	0.69 ± 0.08	0.85 ± 0.05	0.91	0.95
Evoked	0.65 ± 0.2	0.84 ± 0.06	0.62 ± 0.16	0.89 ± 0.04	0.84 ± 0.07	0.81 ± 0.06	0.75	0.91

All values represent Pearson correlation between trial-averaged timecourses of either beta modulation, gamma modulation, or evoked responses, for two subjects (S1 and S2).

### 3.3 Closed-loop operation

**Fig. 3.**
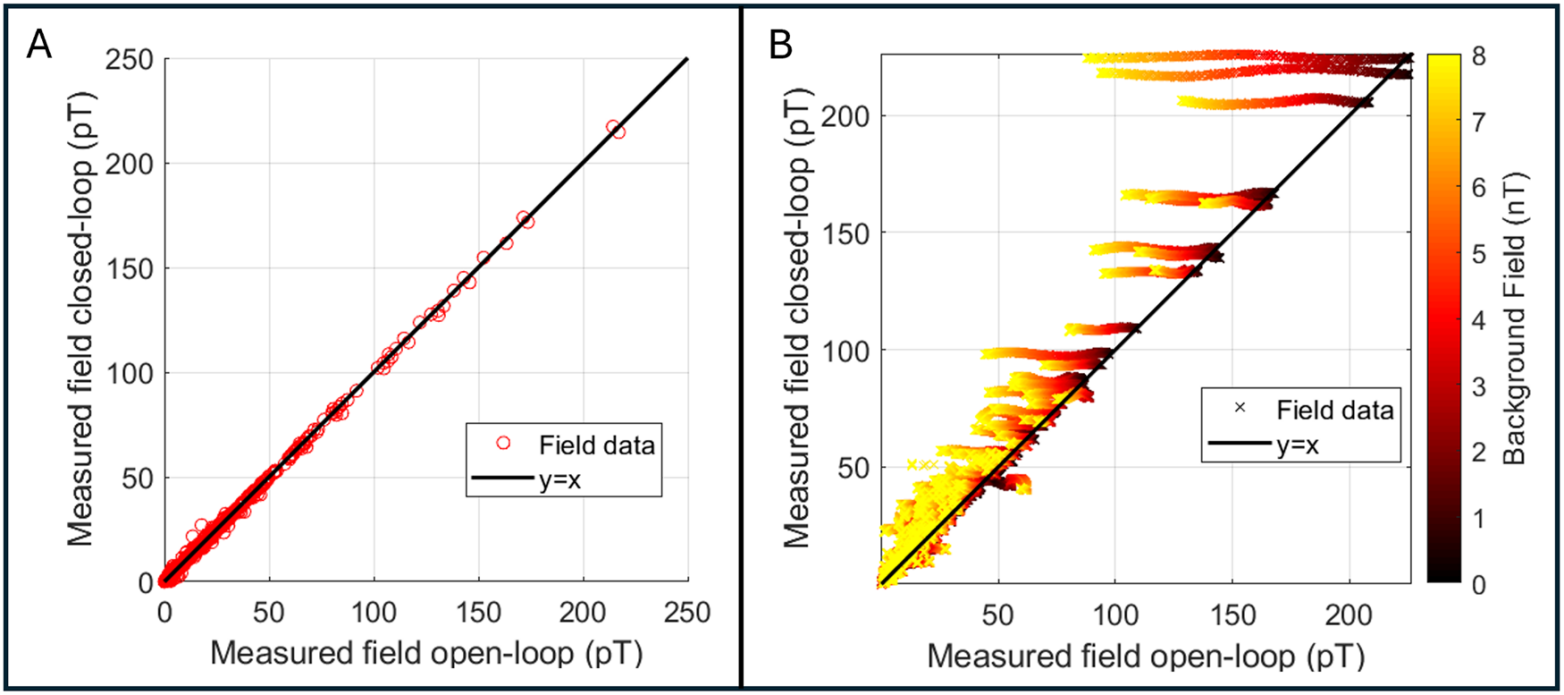
Phantom experiments to characterise closed-loop operation. (A) Measured phantom field amplitudes with zero background field. Measurements in closed-loop mode plotted against equivalent measures in open-loop mode. Note the linear relationship showing that closed-loop operation makes no difference to field measurement in low background field. (B) Phantom fields measured with a varying background. Measures acquired with closed loop on are plotted against equivalent measures in open-loop mode. Here, the background field was allowed to vary between 0 and 8 nT (delineated by the colour of the points). As background field is increased, the open-loop fields decrease, yet the closed-loop fields remain at the same value.

Figure 3 shows the results of our phantom experiments. Panel A shows the amplitude of the phantom field (i.e., the amplitude of the 17 Hz component of the Fourier spectrum) measured using open loop (plotted on the x-axis) versus closed loop (plotted on the y-axis). All data were acquired in zero-background field and the black line represents the line of equality. The linear relationship shows that using closed-loop mode makes no difference to the measurements. Panel B again shows open-loop measures of the phantom field plotted against closed-loop values. However, here the background field was allowed to vary between 0 nT and 8 nT (this is represented by the colour of the data points). As background field was increased, the fields measured in open-loop mode decrease as expected (this is a result of the non-linear OPM response in large background fields—note that the output measure becomes less accurate with increasing field). In contrast, the fields measured in closed loop remain the same regardless of background field (save for a small error, the origin of which is unclear).

To make a quantitative assessment of these effects, we took all the measurements and subtracted the value measured with zero background field (i.e., we removed our best assessment of the true value) for the 10 sensors measuring the largest phantom field. We then computed the difference from zero as an estimate of the error introduced by the non-zero background. In closed-loop mode, this error was 0.5 ± 0.35% (mean ± standard deviation), whilst in open-loop mode it was 14.2 ± 14.9 %, for a range of background fields of 0 nT–8 nT.

**Fig. 4.**
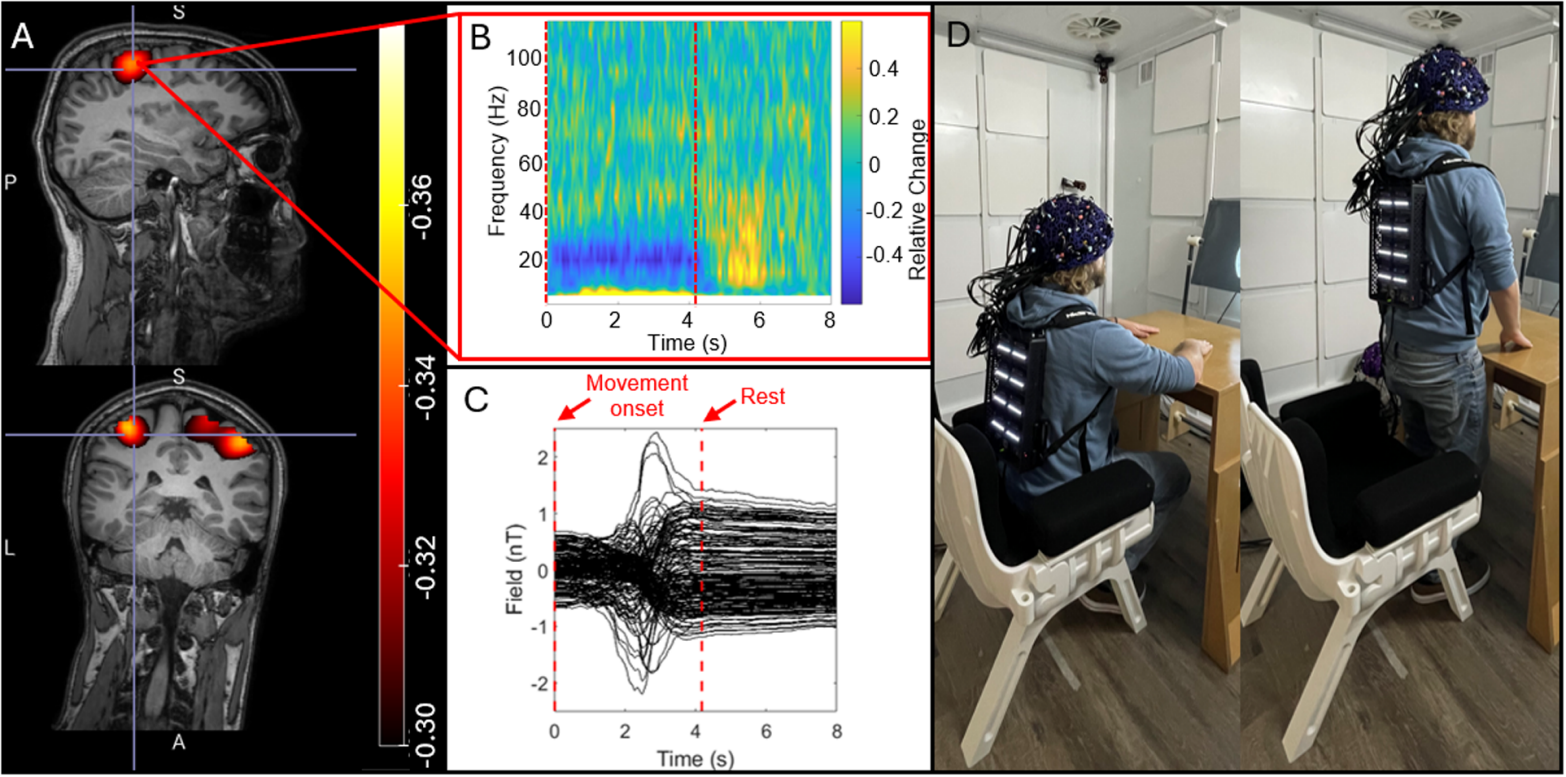
Sitting-to-standing task: (A) The spatial signature of beta modulation induced by the task. (B) The raw magnetic fields measured by the channels, showing that sensors travelled through a ~2 nT background field as the participant moved from sitting to standing. (C) A TFS from sensorimotor cortex showing the time frequency evolution of neural oscillations. (D) A re-enactment of the task to demonstrate range of motion.

Figure 4 shows the results of our sitting-to-standing task. Figure 4A and B show pseudo-T-statistical images of beta modulation and a TFS extracted from the peak in primary sensorimotor cortex, respectively. The largest beta modulation was localised to the bilateral sensorimotor regions, extending from the hand area medially to the areas responsible for leg movement (recall that the task involved finger movement whilst standing up, so this is to be expected). The TFS showed clear beta band desynchronisation in the first 4 s of each trial whilst the subject was moving. Figure 4C shows the raw magnetic field data measured by the sensors. Most sensors show a background field shift, generated by movement, of >1.5 nT—this is more than the dynamic range of the sensors when running in open-loop mode. Despite these large field shifts, the sensors maintain operation. While these measurements would be possible with the sensors running in open-loop mode, the accuracy of the signals would be significantly impeded by both gain and CAPE errors (Borna et al., 2022).

### 3.4 Cross-site comparison

**Fig. 5.**
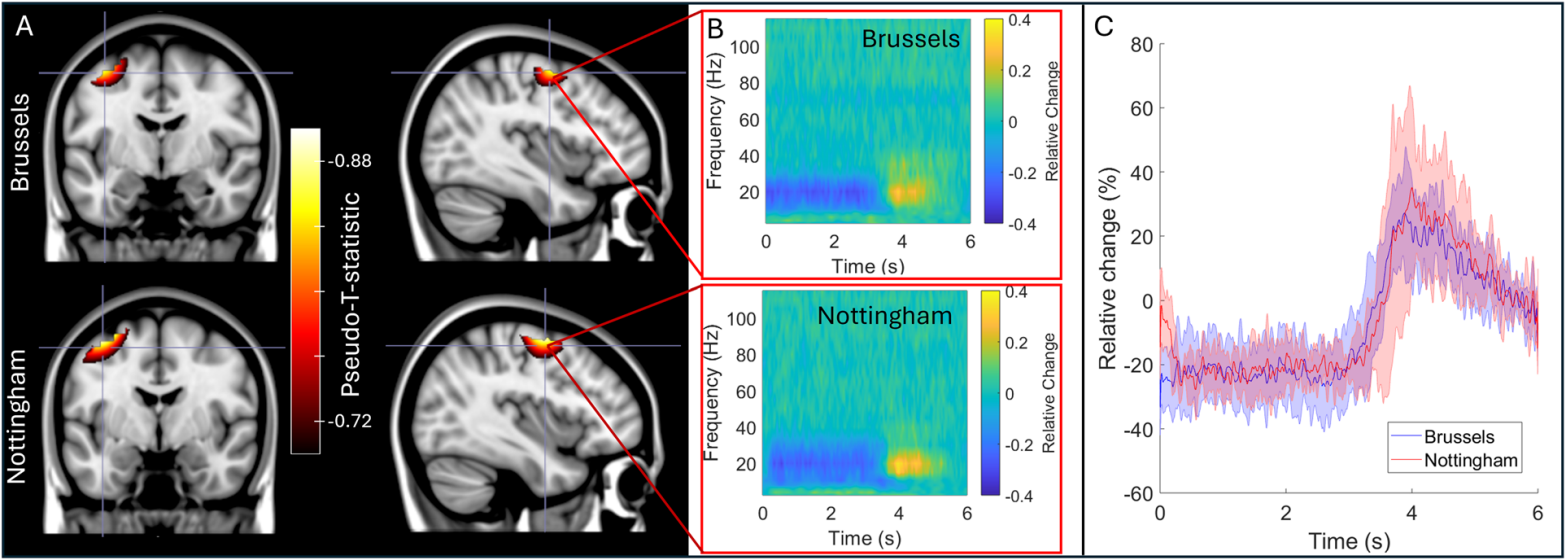
Cross site comparison: (A) Pseudo-T-statistical images for the group average beta band effects at the two sites. In both cases, the upper panels show data acquired at the Brussels site and the lower panels show the Nottingham site. (B) TFSs extracted from the image peaks. (C) Trial-averaged reconstructed timecourses filtered to the beta band, extracted from the image peaks.

Figure 5 shows the results of our cross-site study where the IM system was transported between laboratories in Brussels and Nottingham. Figure 5A shows pseudo-T-statistical images of beta modulation induced by finger movement. The upper panels show Brussels data and the lower panels show Nottingham data—all results are averaged across five subjects at each site. The TFSs in panel B show the time frequency dynamics at the image maxima. Peaks in the averaged images were separated by 1 mm and temporal correlation was 0.96. For direct comparison, Figure 5C shows the envelope of oscillatory amplitude in the beta band, averaged over trials and subjects. Note the high level of similarity.

### 3.5 Concurrent OPM-MEG/EEG

**Fig. 6.**
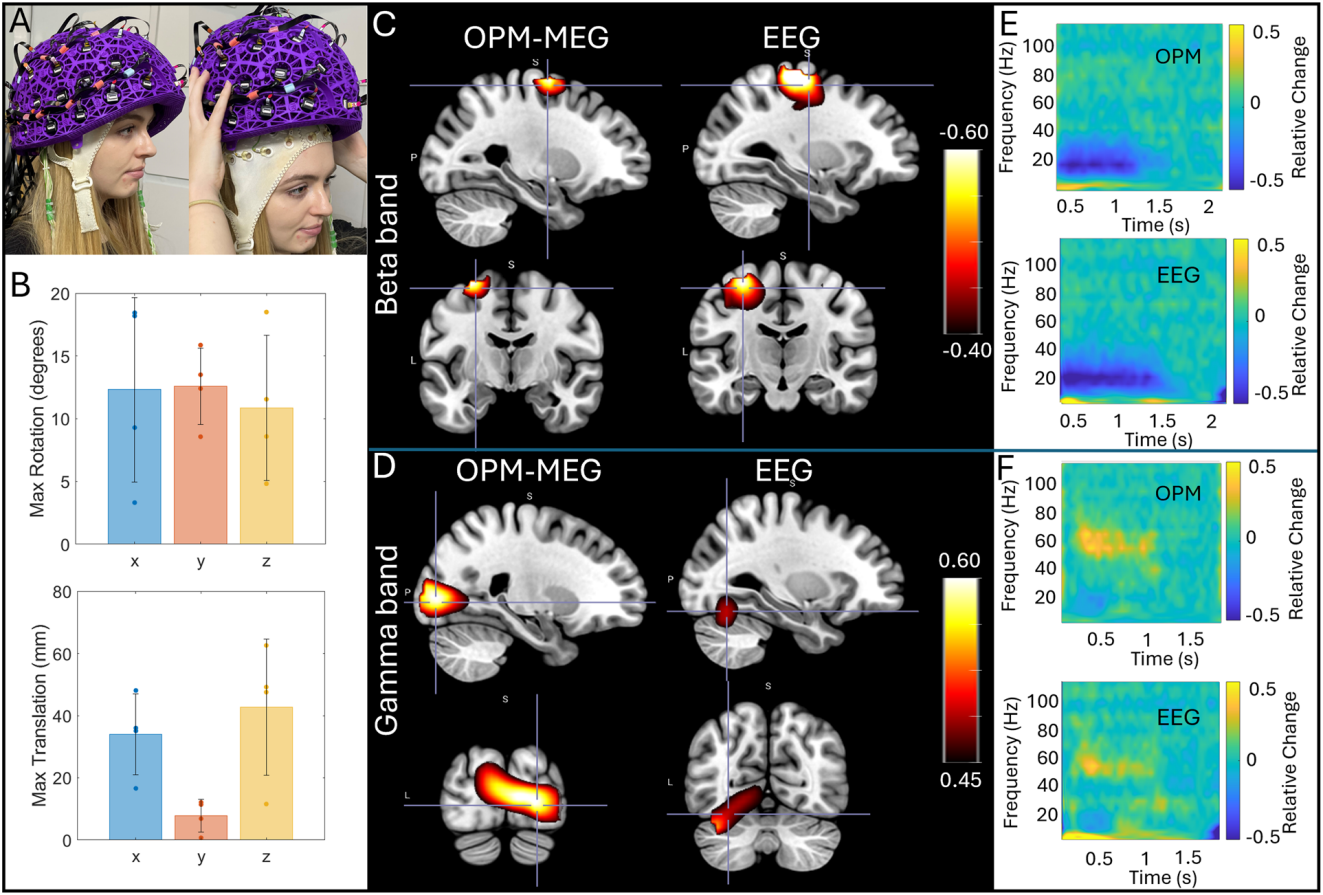
Concurrent OPM-MEG/EEG: (A) A participant wearing an EEG cap and OPM-MEG helmet. (B) Data were recorded during natural head movements: the maximum translations and rotations made by the subjects across the experiments are shown. Bars represent mean across subjects; data points show values for each individual subject. (C) and (D) show group average beta and gamma effects, respectively. In both cases, pseudo-T-statistical images and associated TFSs (from the minima for beta (E) and a central point in the visual cortex for gamma (F)) in those images are shown for EEG and MEG. All data were recorded in the presence of movement. The static case is shown in Supplementary Figure S3.

Finally, Figure 6 shows the results of our concurrent OPM-MEG/EEG experiments. Here five people took part in the experiments, however, averages are shown for four participants since the EEG recording failed in one. Figure 6B shows the extent of the natural movements carried out by subjects during the scans; bar charts show maximum translation (bottom) and rotation (top); the bars show the mean across subjects, and the individual data points show results for the four subjects. Figure 6C and D show concurrently acquired OPM-MEG and EEG data averaged across subjects. Panel C shows the beta band modulation during finger movement and panel D shows gamma modulation by the visual stimulus. In both cases, pseudo-T-statistical images and TFSs are included. As expected, EEG and OPM-MEG show similar effects; both allow recording of beta and gamma oscillations showing the viability of concurrent recordings. However, whereas localisations for OPM-MEG are as expected (primary motor and visual cortices for the beta and gamma effects, respectively), the localisations do not appear as accurate for the EEG data—this will be discussed further below.

## 4 Discussion

Our aim was to demonstrate a new OPM-MEG system with integrated and miniaturised electronics and test its viability for assessment of human electrophysiological function. Our primary demonstration saw the new IM system used multiple times in two subjects, to provide a comparison with an established device, which has previously been validated (Boto et al., 2022; Rea et al., 2022; Rier et al., 2023, 2024), including against conventional MEG (Boto et al., 2021; Hill et al., 2020; Rhodes et al., 2023). The results obtained with the two systems showed striking spatial and temporal consistency. Spatially, the pseudo-T-statistical images showed good agreement between systems (see Fig. 2; Supplementary Fig. S1). Quantitatively, for movement-related beta modulation, the peak locations were within ~10 mm between systems. For gamma and evoked responses, we saw higher spatial discrepancies (~20–30 mm), but these values can be explained. For example, in the gamma band, the centrally presented visual stimulus activates the visual cortex across both hemispheres, and the location of the peak can appear on either side of the calcarine fissure, depending on factors such as gaze position. In the case of subject 1, the peak activity tended to be in the left hemisphere for the RM system and the right hemisphere for the IM system (see Supplementary Fig. S2); this contributed to increased distance between peaks (shown in Table 1). However, when taking into account the images themselves, they show similarity; for example, when thresholding the images at 75% of their maxima, there is clear overlap between the regions delineated (see Supplementary Fig. S4). Similarly for the evoked response, activity can be localised to primary visual and fusiform regions, and this contributes to increased spatial variation. Most importantly, however, in beta, gamma, and evoked measurements, there is good consistency between the images themselves. In addition, the distances between peaks measured between the two systems did not differ significantly from the within-system consistency. Source timecourses were highly reproducible across the two systems with an average correlation of >0.75 for individual runs, and >0.9 for averages of multiple runs in the same subject. Overall, these results show that the two systems provide equivalent performance. Importantly, not only does this validate the IM device, but also shows that the magnetic interference generated by the backpack-mounted electronics inside the MSR is well controlled by HFC (Tierney, Alexander, et al., 2021) and beamforming (Brookes et al., 2021).

The addition of closed-loop sensor operation is a significant technical milestone. The dynamic range of OPMs is low (±1.5 nT), and maintaining an environment where fields are limited to this range is challenging. Firstly, even inside magnetic shields, environmental changes in magnetic field over time (e.g., caused by local infrastructure) can be much larger than the dynamic range of the OPMs (e.g., Hill et al., 2022). Secondly, most MSRs have a temporally static field ranging between 3 and ~30 nT. These fields are typically nulled within each OPM using on-board sensor coils at the start of an experiment and so have no effect on measurements, if OPMs remain stationary. However, if the head is allowed to move in this static field, then the OPMs “see” a field that changes in time and can be taken outside their dynamic range.

The effects of both environmental field shifts and subject movement are further complicated by the orientation of the background field relative to the sensor: Let us assume we want to measure a field of interest, $B_{x}$, oriented in the x-direction. However, the measurement must be made in a background field described by the vector $\left[B_{bx},B_{by},B_{bz}\right]$. Ideally, the sensor output along the x-direction would be $B_{x}+B_{bx}$ (i.e., the field of interest plus the background); indeed this is the case for low values of $B_{bx}$, $B_{by}$ and $B_{bz}$ (when the sensor is operating within its dynamic range). However, when background fields become large, then the presence of a background field along the measurement direction, $B_{bx}$, will cause the OPM response to become non-linear, which manifests as a drop in sensor gain, meaning that the measurements of both $B_{x}$ and $B_{bx}$ will be smaller than their true values. If $B_{by}$ and $B_{bz}$ are large, this also causes a drop in OPM gain (again the measurement of $B_{x}$ diminishes); in addition, changes in $B_{by}$ and $B_{bz}$ over time can manifest as a change in $B_{x}$—this is known as cross-axis projection error (CAPE) (Borna et al., 2022). The ability to make closed-loop measurements, where measured fields at the vapour cell are compensated in real time by equal and opposite fields generated by the on-board sensor coils, offers a solution to this problem by making the sensor robust to changes in background field. However, because background fields in any orientation cause inaccuracies, complete three-axis closed-loop operation is critical.

Here, we assessed closed-loop sensor operation in two ways. Firstly, we operated sensors in zero background field, in closed and open loops. The linear relationship shown in Figure 3A suggests that closed-loop operation made no difference to the sensor output (within the 0–200 pT range of fields that we employed). (Additional noise measurements, see Supplementary Fig. S1, showed there was no effect on noise floor). Our second phantom experiment (Fig. 3B) showed that, when background fields were applied, open-loop operation becomes inaccurate, with (in this particular experiment) sensor outputs dropping by ~14% (on average over the 10 OPM channels measuring the largest phantom fields). However, when operating in closed-loop mode, the equivalent sensor error is within 0.5%. Importantly, the background field (which was oriented vertical relative to the MSR) would have intersected sensors with a number of different orientations since the sensors were positioned in a helmet, meaning that the triaxial nature of closed-loop operation was tested. The result, therefore, shows that three-axis closed-loop operation can effectively linearise the response of an OPM in background fields of varying orientation up to and including 8 nT. In theory, the same approach can be used to reach even higher background field values (limited only by the range of field that can be generated by the on-board sensor coils—±50 nT). This should be a topic of future system development.

A concern for all OPM array designs is the potential effect of cross talk, that is, whether measurements at sensor “A” are affected by a second sensor, “B”, in proximity. The mechanism of cross talk for OPMs involves the magnetic fields generated by the on-board coils spreading across sensors. In open loop, the modulation field (which determines the orientation along which the measurement takes place) from sensor B can constructively interfere with the modulation field at sensor A, changing the latter’s gain and orientation specificity. In closed loop, OPMs are driven with negative feedback and so the applied fields at sensor B, which must cancel measured brain activity, could spread to sensor A. This might mean that the measured brain activity at sensor A is either partially cancelled out, or amplified, by cross talk from B. Here, we quantified this effect (see Appendix A1). We found that for the present array design, on average the biggest cross talk that a sensor experiences was 0.7 ± 0.3% and the absolute maximum cross talk between any pair of sensors was 1.6%. This was deemed acceptable for the measurements made. However, results also showed that (as would be expected) cross talk changes significantly with distance between sensors, and so in future studies using high-density arrays, this effect must be taken into account. This is the case for both open- and closed-loop sensor operation.

One of the biggest advantages of OPMs over conventional MEG is that the sensors move with the head, enabling movement during a scan. This was shown here via two experiments—the sitting-to-standing task, where a participant transitioned from being seated to standing (Fig. 4), and the concurrent EEG measurements, where participants made natural head movements. This type of investigation has significant utility—for example, the sitting-to-standing task is used widely in clinical assessments of lower limb function, mobility, and fall risk across a range of conditions (e.g., Nocera et al., 2013). Similarly, children find it hard to keep still in conventional scanning environments, as do patients who may often be nervous, in pain, or even undergoing seizures (Feys, Corvilain, Van Hecke, et al., 2023; Hillebrand et al., 2023). The ability to scan people whilst moving, therefore, offers a more effective environment to gather MEG data. The introduction of our IM system offers two improvements in this area: Firstly, the new backpack-mounted electronics reduces the cabling between the backpack and the outside of the shielded room. The total cable weight for the RM system is 14 kg, which is reduced to 1.3 kg for the IM system, though of course not all this weight is supported by the subject. When standing, the subject has to bear the weight of ~1 m cabling (which falls from the backpack to the floor)—this was estimated (using a Newton meter) at 4 kg for the RM system and 300 g for the IM system, a reduction by a fact of 13. This makes naturalistic experiments where subjects are standing or walking much more practical. In addition to weight, there are only 2 cables to the IM system which can be easily managed (distinct from 64 cables for the RM system). Secondly, closed-loop operation means that sensor output remains linear even when the sensors move through a large background field; this enables sensors to keep working when the subject moves (as demonstrated in Fig. 4). In previous demonstrations of free movement (Holmes et al., 2018, 2023; Rea et al., 2022), sensor linearity has been enabled by the use of large coils—either mounted on planes each side of the subject, or within the MSR walls—which create a zero field volume enclosing the head. In principle, closed-loop operation offers an alternative to use of such coils. However, it is important to note that larger coils not only linearise sensor output, but because they zero the field across the entire head volume, they also reduce artefacts caused by movement. Closed-loop operation linearises output but *does not remove artefact*. This means that large scale coils are still a critical requirement for OPM-MEG instruments. However, it is worth noting that the increased accuracy in signal measurement from closed-loop operation may allow better characterisation and, therefore, removal of artefacts from data.

As part of the evaluation of our IM system, we exploited the compact and lightweight nature of the system by transporting it between two laboratories in different countries. Practically, this proved to be relatively straightforward—the system fitted into two suitcases and was easily transportable between sites. Such portability is somewhat tempered by the requirement for an MSR at both sites. Nevertheless, it offers new opportunities: For example, it becomes possible to design and build a single optimised array and scan individuals at multiple sites. This would be of significant utility—for example, to expand the number of patients who could be scanned in a single study by taking the same system to the patients. Equally, one can imagine shipping a system to more clinically oriented sites where it could be used to scan patients who require constant medical attention. Perhaps most interestingly, the high level of portability and the low infrastructure requirements of our IM system make it ripe for deployment on a mobile platform (i.e., an OPM-MEG system in a truck). This would have multiple uses—for example, it could be deployed as a facility that could visit multiple epilepsy clinics—enabling patients at multiple sites to benefit from MEG (Rampp et al., 2019) whilst minimising cost. It could also enable deployment of OPM-MEG in “field trials”, for example, at military training establishments or sports grounds to monitor concussion (Rier et al., 2021). Finally, a mobile platform could be used to gather large data sets from multiple geographic locations—including varying socioeconomic regions—something that is challenging when scanners are based in universities. In sum, the highly portable nature of the IM system offers new opportunities that are not accessible using conventional (cryogenic) MEG technology.

Figure 6 shows that our IM system can be used simultaneously with EEG to gather multi-modal electrophysiological data. This is not the first time OPM-MEG/EEG has been carried out (e.g., Boto et al., 2019; Feys, Wens, et al., 2023; Seedat et al., 2023), and these previous studies have shown that the presence of OPMs does not impact significantly the quality of the EEG data, or vice-versa. Our aim here was simply to demonstrate that concurrent recordings are possible. This is important for future clinical use since the acquisition of multi-modal data means clinical neurophysiologists can acquire EEG—which they are highly familiar with—at the same time as OPM-MEG data, which offers significant advantages in terms of spatial accuracy. As such, it will allow clinicians to relate EEG features which are detected from specific EEG montages to the high-density, source-localised OPM-MEG signals which will provide a bridge for translation of this new technology into the clinic. It was not our intention to directly compare OPM-MEG and EEG; nevertheless anecdotally, results from both modalities show clear beta and gamma band responses, even during head movement. However, whereas OPM-MEG consistently placed the gamma response in primary visual cortex, the EEG localisation placed it lower, in the cerebellum—this is likely a result of the challenges in EEG forward modelling—including inaccurate knowledge of conductivity values of the tissues in the head (Baillet, 2017).

Finally, from a practical point of view, the IM system performed well. In previous OPM-MEG systems, robustness has been a key concern—in particular, the number of channels that are lost in a measurement. Here, across 32 experiments using our IM system, we lost (on average) 3 ± 5 channels. Where we did lose channels, the reason was typically a connection between the sensor head and ribbon cable. Sensor heads are connected using a latch which clamps down on the ribbon cable, making an electrical connection. This necessitates minimal tolerance when manufacturing the cables, since even small changes in cable thickness can make the latch connector loose, and consequently the connection temperamental (this was also the likely reason for the marginally increased empty room noise in the IM system). This is something that should be altered in future generations of this system. Despite this minor limitation, the IM system performed well. The set-up time for 64 sensors was typically around 3 min—this includes the time to heat the vapour cells and lasers, lock their temperature with PID controllers, and optimise all sensor parameters, zero the field within each cell, calibrate the sensor, and turn on the closed loop. Each OPM sensor head has slightly different properties, meaning that control parameters must be optimised on a per sensor basis (much like superconducting quantum interference devices (SQUIDs) must each be individually tuned in a conventional MEG system). In the IM system, because these parameters are optimised and set on sensor start-up, sensor heads can be swapped easily with no requirement for anything other than a sensor restart following a swap. This is a significant practical advantage when running the system, adding further modularity to the design.

## 5 Conclusion

We reported a new OPM-MEG system design with miniaturised and integrated electronic control, a high level of portability, and significantly improved dynamic range. We have demonstrated that this instrumentation offers equivalent measures of induced and evoked neuro-electrical responses to stimuli compared with an established instrument, and that it offers improved dynamic range, up to and including 8 nT shifts in background field. We have shown that the system is effective in gathering data during participant movement (including a sitting-to-standing paradigm) and that it is compatible with simultaneous EEG recording. Finally, we demonstrated portability by moving the system between two laboratories. Overall, our new system represents a significant step forward for OPM-MEG and offers an extremely attractive platform for next generation functional medical imaging.

## Supplementary Material

Supplementary Material

## Figures and Tables

**Appendix Fig. A1:**
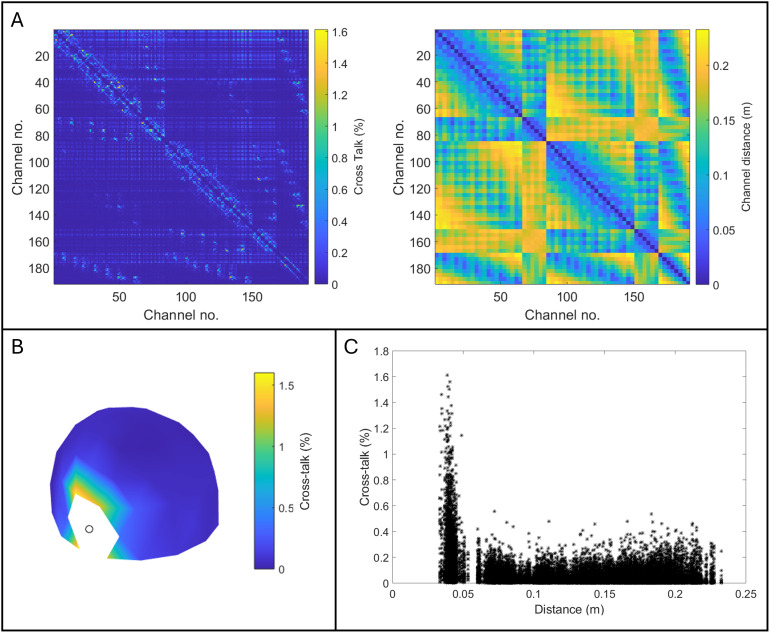
Cross-talk measurements: (A) Cross-talk matrix (left) and Euclidean distance between channels (right). (B) Topography of cross talk from a single sensor (sensor location marked by the black circle). (C) Cross talk plotted against distance between sensors.

## Data Availability

All code was custom developed in-house using MATLAB and is available from https://github.com/HollySchofield/Schofield2024_novel_robust_platform_OPM. This repository also contains a link to the data, which were acquired by authors.

## Appendix A1 Cross-Talk Measurements

Cross talk in OPM-MEG manifests as the spread of magnetic fields, generated by on-board sensor coils, to nearby sensors. This is a significant concern for all OPM arrays and is of particular interest in closed-loop operation, since the on-board coils are constantly applying fields (as part of a negative feedback loop) to maintain zero field at the OPM sensor cell. In closed-loop operation, the applied fields must mimic measured brain activity (as well as sources of interference). It is, therefore, conceivable that that cross talk between sensors operated in closed-loop mode could cancel out, or amplify, brain activity (and interference) measured at a nearby sensor. Here, we sought to quantify this effect.

### A1.1 Method

All 64 sensors were placed in the 3D-printed helmet used for the measurements described above. The helmet was placed at the centre of the shielded room; the room walls were demagnetised and matrix coil currents applied to null background fields (i.e., the same procedure as the measurements in the main paper). Following this, all sensors were calibrated. For each sensor, an 8-s sinusoidal pulse was applied simultaneously to the X, Y, and Z oriented on-board sensor coils, at 23 Hz, 29 Hz, and 37 Hz, respectively. These pulses were applied to all sensors sequentially and data were recorded throughout. The experiment lasted 512 s.

Following collection, data were segmented using the start and end of the pulse, to define 64 “trials”; here, a single trial represents the three coils at one sensor being energised. For each trial, a Fourier transform of the data was computed at all 192 measurement channels, and the spectral amplitudes at 23 Hz, 29 Hz, and 37 Hz were calculated. These values were normalised by the spectral amplitude of the signal at the sensor being energised; this resulted in a quantification of cross talk, expressed as a percentage. That is, assuming we are applying fields to channel “A”, then a cross-talk value of 10% at channel “B” would mean that channel “B” “sees” 10% of the field amplitude being applied to channel “A”. Performing this analysis for all axes of all channels resulted in a 192 x 192 matrix of cross talk between all possible channel pairs. Note that two bad channels were removed from the analysis.

### A1.2 Results

Appendix Figure A1A (left panel) shows the full cross-talk matrix; for reference the right hand panel shows Euclidean distances between the interacting channels. Appendix Figure A1B shows the spatial topography of cross talk across the helmet for a single sensor (this sensor was selected because it showed the highest cross talk). Appendix Figure A1C shows the magnitude of cross talk for all channel pairs, plotted as a function of distance between sensors. Across all possible pairs of channels, the maximum measured cross talk was 1.6% (at a sensor separation of 38 mm). Cross talk fell rapidly with distance, as would be expected given that the coils are effectively current dipoles. Given these relatively low values, we suggest that, for the present array, cross talk is not affecting our measurements significantly. However, in future studies where sensors may be operated in proximity, cross talk may need to be accounted for.
