# Supplementary material for: A novel, robust, and portable platform for magnetoencephalography using optically-pumped magnetometers
Source: Imaging Neurosci (Camb). 2024 Sep 25;2:imag-2-00283. doi: 10.1162/imag_a_00283 (PMC11533384; doi:10.1162/imag_a_00283)
Supplement: Supplementary Material [file imag_a_00283-supp.pdf]

SUPPLEMENTARY INFORMATION

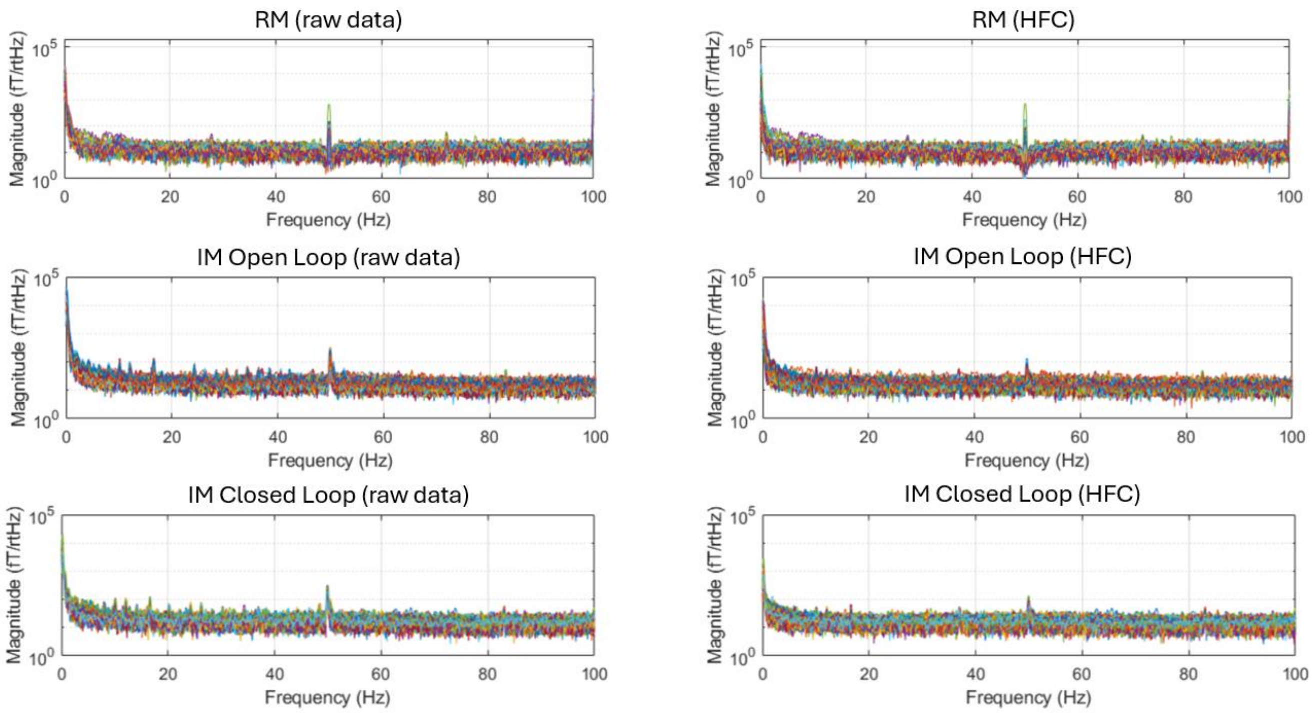

Figure S1: Representative noise spectra (with (right) and without (left) HFC) for the RM system (top) the IM system in open loop (middle) and the IM system in closed loop (Bottom)

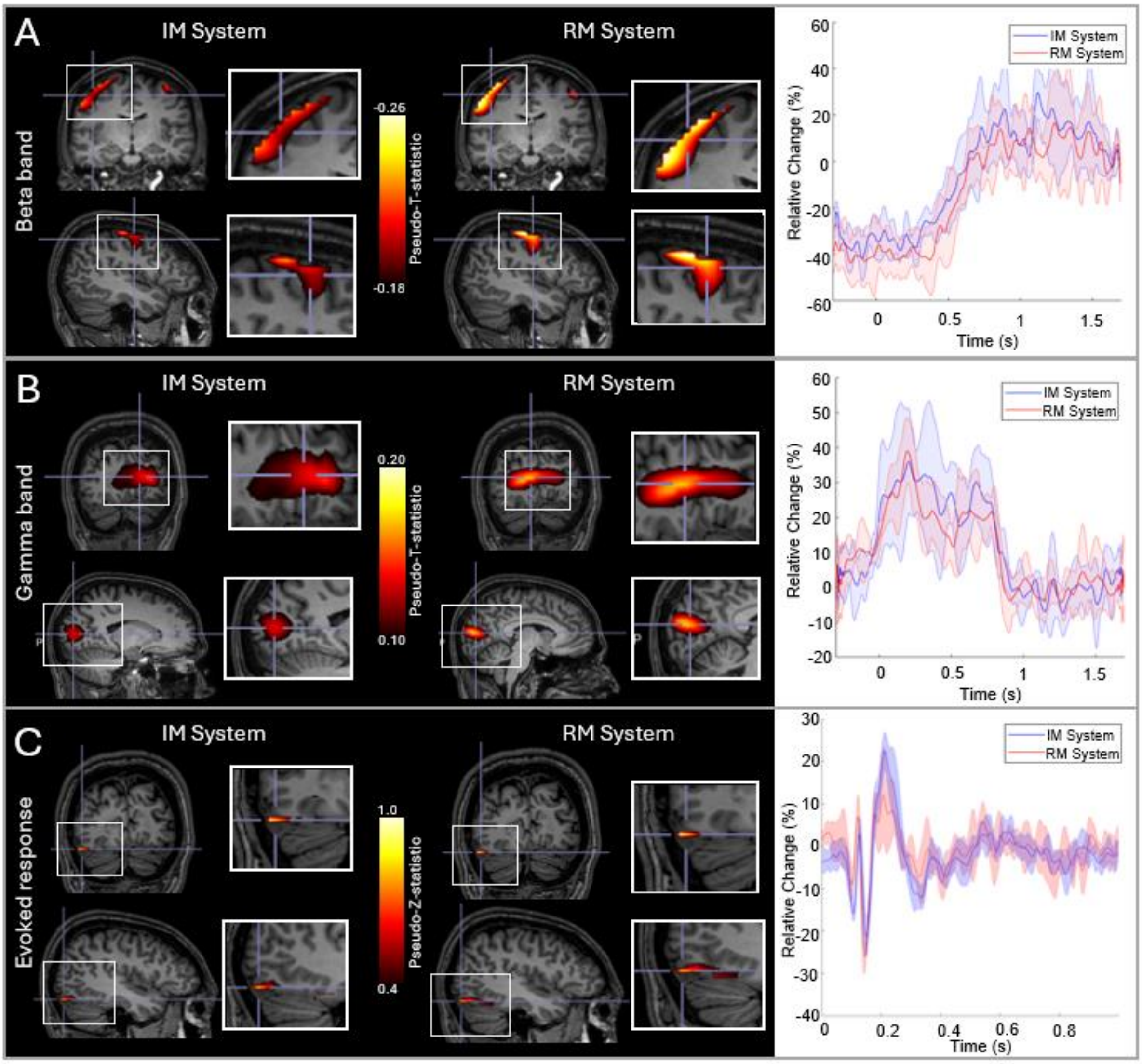

Figure S2: RM and IM system comparison for S1: *Layout equivalent to Figure 2, but results shown for Subject 1 (S1).*

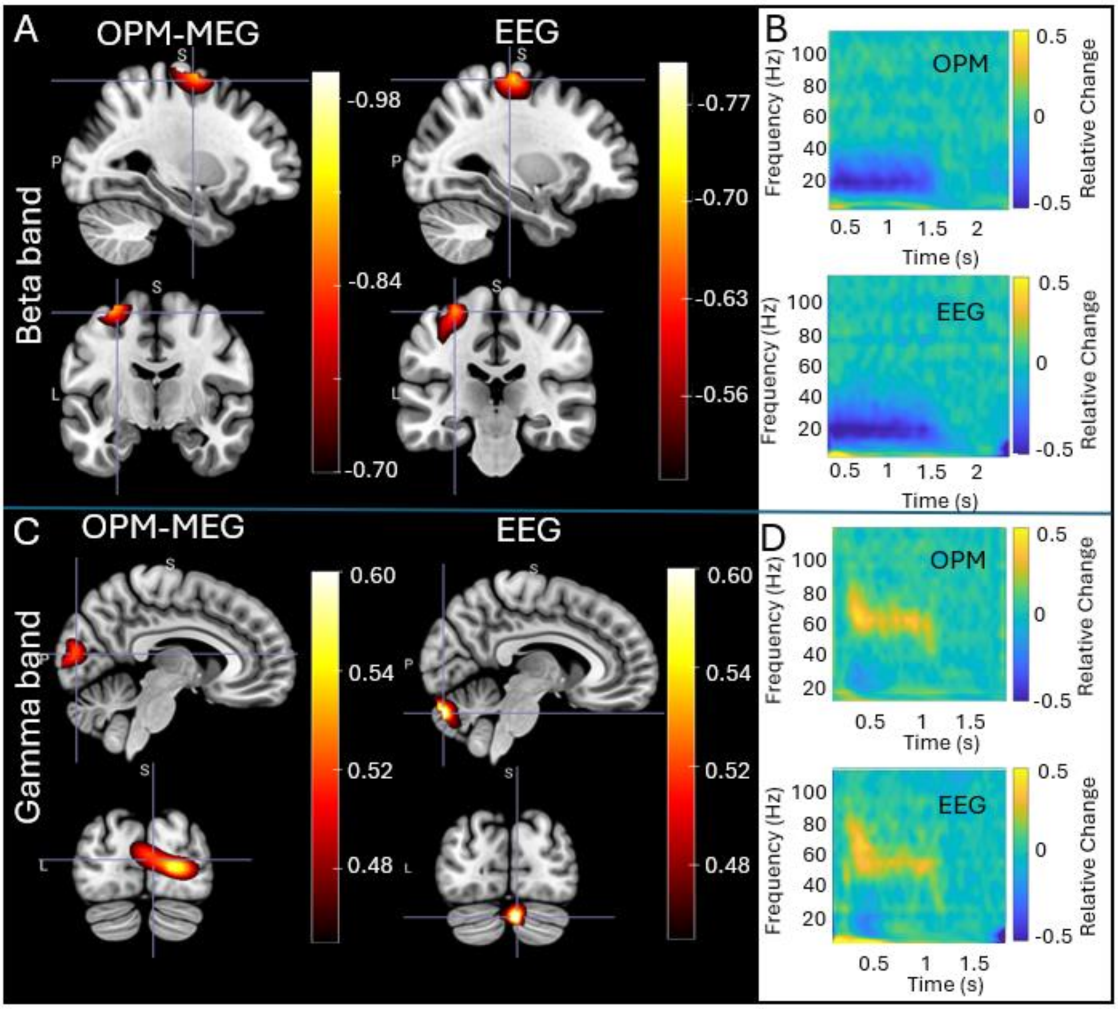

Figure S3: Concurrent OPM-MEG/EEG: *Same as Figure 6 but in the static case (i.e. no head motion).*

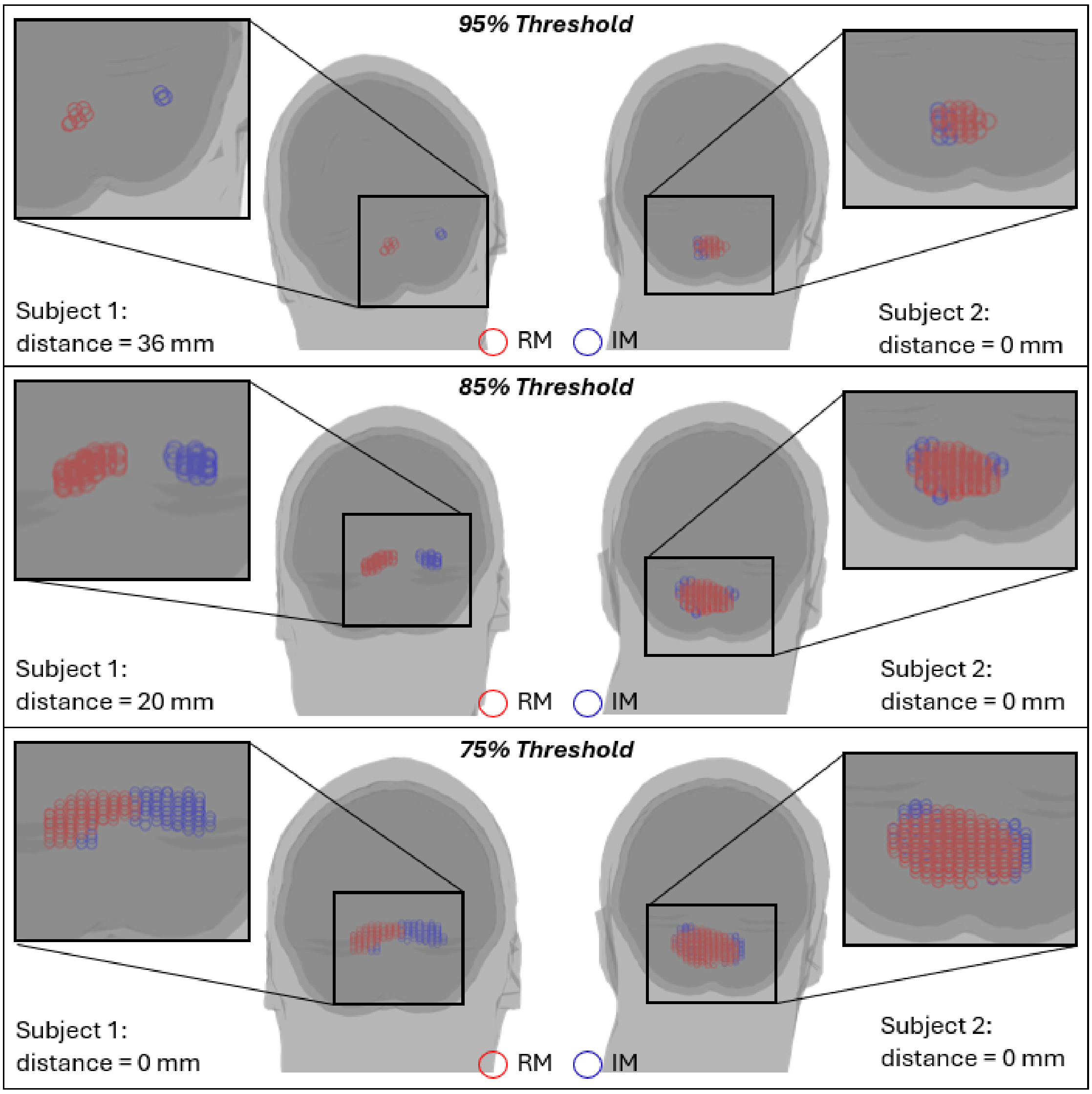

Figure S4: Additional spatial analyses. *Here, pseudo-T-statistical images have been thresholded at either 95% (top panel) 85% (centre column) or 75% (bottom column) of their maximum to give a cloud of points depicting maximum T-statistics. In all three panels the left-hand image shows the spatial distribution of gamma modulation for subject 1. The right-hand panel shows the same thing for subject 2. In both cases, red circles show the point cloud for the RM system and the blue circles shows the point cloud for the IM system. Inset images show zoomed version and values represent the distance between the closest points in the two point clouds. Notice that the distances fall to zero when thresholding images at 75%.*
